# Characterization of DNA polymerase β from *Danio rerio *by overexpression in *E. coli *using the *in vivo*/*in vitro *compatible pIVEX plasmid

**DOI:** 10.1186/1475-2859-10-84

**Published:** 2011-10-21

**Authors:** Tomomi Ishido, Naoshi Yamazaki, Mitsuru Ishikawa, Ken Hirano

**Affiliations:** 1 Health Research Institute, National Institute of Advanced Industrial Science and Technology (AIST), Takamatsu, Kagawa 761-0395, Japan; 2 Faculty of Pharmaceutical Sciences, University of Tokushima, Sho-machi, Tokushima 770-8505, Japan

## Abstract

**Background:**

Eukaryotic DNA polymerase β (pol β), the polymerase thought to be responsible for DNA repair synthesis, has been extensively characterized in rats and humans. However, pol β has not been purified or enzymatically characterized from the model fish species *Danio rerio *(zebrafish). We used the *in vitro*/*in vivo *dual expression system plasmid, pIVEX, to express *Danio rerio *pol β (*Danio *pol β) for biochemical characterization.

**Results:**

*Danio *pol β encoded by the *in vitro*/*in vivo*-compatible pIVEX plasmid was expressed in *E. coli *BL21(DE3), BL21(DE3)pLysS, and KRX, and *in vitro *as a C-terminal His-tagged protein. *Danio *pol β expressed *in vitro *was subject to proteolysis; therefore, bacterial overexpression was used to produce the protein for kinetic analyses. KRX cells were preferred because of their reduced propensity for leaky expression of pol β. The cDNA of *Danio rerio *pol β encodes a protein of 337 amino acids, which is 2-3 amino acids longer than other pol β proteins, and contains a P63D amino acid substitution, unlike mammalian pol βs. This substitution lies in a hairpin sequence within an 8-kDa domain, likely to be important in DNA binding. We performed extensive biochemical characterization of *Danio *pol β in comparison with rat pol β, which revealed its sensitivity to metal ion activators (Mn^2+ ^and Mg^2+^), its optimum salt concentration (10 mM KCl and 50 mM NaCl), alkaline pH optimum (pH 9.0), and low temperature optimum (30°C). Substituting Mn^2+ ^for Mg^2+ ^resulted in 8.6-fold higher catalytic efficiency (*k*_cat_/*K*_m_).

**Conclusions:**

Our characterization of pol β from a model fish organism contributes to the study of the function and evolution of DNA polymerases, which are emerging as important cellular targets for chemical intervention in the development of anticancer agents.

## Background

In eukaryotic cells, nuclear DNA replication and repair relies on five species of DNA polymerases (pols): α, β, γ, δ and ε [1-3]. Additional DNA pols have been discovered, namely, pol λ, μ, ζ, η, θ, ι,κ, σ and REV1 [4,5]. These pols have emerged as important cellular targets for chemical intervention in the development of anticancer agents [6,7]. Of these pols, pol β is thought be involved in DNA repair synthesis [8]. This enzyme belongs to family X, which includes pol λ, μ, and terminal deoxynucleotidyltransferase (TdT). Pol β is the smallest polymerase in family X. Other members of the family have a nuclear localization signal (NLS), a BRCA1 C-terminal (BRCT) domain, and a proline rich region. Mammalian pol β is a single polypeptide of 39 kDa, comprising 335 amino acids forming two domains: an 8 kDa N-terminal domain and a 31 kDa C-terminal domain, connected with a protease-hypersensitive hinge region. The former domain possesses a template-binding function and a dRPase activity to remove the 5'-deoxyribose phosphate, the latter domain possesses DNA polymerase activity. Biochemical studies have shown that pol β releases the 5'-terminal dRP residues from the aprinic/apyrimidinic sites and fills short gaps of one to six nucleotides. These activities indicate that pol β functions in base excision repair (BER), which is a major pathway for repairing modified bases in DNA [9].

Both rat and human pol β genes have been cloned, the crystal structures were determined, and an active recombinant enzyme has been overexpressed in *Escherichia coli *[10]. However, only a few papers on the properties of pol β from lower vertebrates, particularly from the fish kingdom, such as *Oncorhynchus masou *(cherry salmon) [11] and *Xiphophorus maculaus *[12], have been reported.

*Danio rerio *(zebrafish) has long been used as a model organism in the fields of molecular genetics and developmental biology of vertebrates [13]. *D. rerio *constitutes a powerful animal model, largely because of their small size, optical clarity, fecundity, rapid development, and large arsenal of readily available genetic tools [14]. The *D. rerio *model system is amenable to whole-genome forward genetic approaches, which facilitates experimental manipulation and allows the direct observation of tissue formation and organogenesis *in vivo *[15]. Thus, research using *D. rerio *has allowed advances in fields such as developmental biology, oncology, toxicology, and regenerative medicine [16-18]. However, the purification and enzymatic characterization of pol β from *D. rerio *has not been reported.

The pIVEX plasmid, which is optimized in the Rapid Translation System (RTS) as cell-free protein expression system, is a useful compatible vector for both *in vivo *and *in vitro *expression systems based on *E. coli*, pIVEX permits comparison between cell-free and bacterial expression from the same genetic construct [19]. The pIVEX vector system is particularly useful for (i) bacterial expression after cell-free expression for scale-up of protein expression and solution of problems such as poor protein yields or expression results, and (ii) cell-free expression when the recombinant protein cannot be expressed in bacteria cells. In fact, in the present study, we found pIVEX carrying the *Danio rerio *pol β (*Danio *pol β) cDNA causes growth inhibition of BL21(DE3)pLysS cells before expression induction, which contrasted with other proteins that were reported to be expressed in the pIVEX vector system using BL21(DE3)pLysS cells [19,20]. Thus, the determination of the correct plasmid/host combination is required for the expression certain proteins.

In the present study, we report the overproduction and enzymatic characterization of *Danio *pol β using the pIVEX plasmid in *E. coli*. We revealed that *Danio *pol β was overproduced in the *E. coli *expression host by substituting ordinary BL21(DE3) cells with KRX cells. The enzymatic properties of the purified recombinant *Danio *pol β were examined and compared with rat pol β. The Michaelis constants, *K*_m _and *k*_cat_, and the catalytic efficiencies (*k*_cat_/*K*_m_) for recombinant *Danio *pol β were also determined by pol β-mediated nucleotide incorporation during primer extension.

## Results and Discussion

### Comparison of amino acids sequences among various pol βs

**Figure 1:**
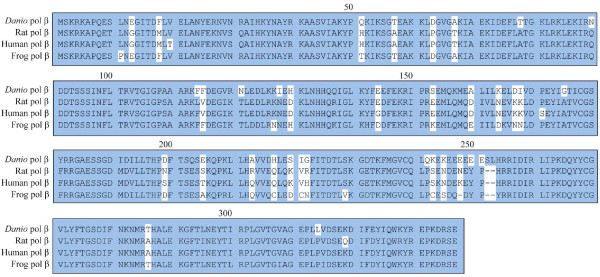
**Comparison of the amino acid sequences of between *Danio rerio *polymerase β and other DNA polymerase βs**. Similar amino acids are shown in blue. The amino acids sequence of ***D. rerio ***pol β is compared with that of rat [Swiss-Prot: P06766], human [Swiss-Prot: P06746], and frog [Swiss-Prot: O57383].

Figure 1 shows a comparison of pol β amino acid sequences from *D. rerio*, frog, rat, and human. The cDNA of *D. rerio *pol β encodes a protein of 337 amino acids, which is two residues longer than the human, rat, calf, or mouse protein (all 335 amino acids) and three residues longer than that of frog (334 amino acids). The polypeptide length of *Danio *pol β was the same as for pol β in another fish, *Xiphophorus maculaus*. The amino acid sequence of *Danio rerio *pol β has 80% sequence identity to the equivalent sequence in rats, 79% to the human protein, and 82% to the frog protein. One notable amino acid difference between the *D. rerio *and the mammalian proteins was the P63D amino acid substitution in *D. rerio*. This difference occurs in the L62-P63-G64-V65-G66 sequence in human and rat pol β, which represents a hairpin sequence within the 8-kDa domain. This region exhibits a high degree of polypeptide backbone motion, which is likely to be important in DNA binding as well as in the formation of the dNTP binding pocket [11]. Other key residues are: D190, D192 and D256 for base pair geometry in active site, and control of binding and placement of catalytic metal ion; Y267 for stabilizing the nucleotide binding pocket and promoting proper alignment of primers, Mg^2+^, dNTP, and other active site residues; and R285 and K282 for hydrogen bonding of nascent base pairs to template nucleotides [12,21]. However, no differences in these other key residues were not observed in *Danio *pol β.

### Growth rate of *E. coli *cells transformed with pIVEX

**Figure 2:**
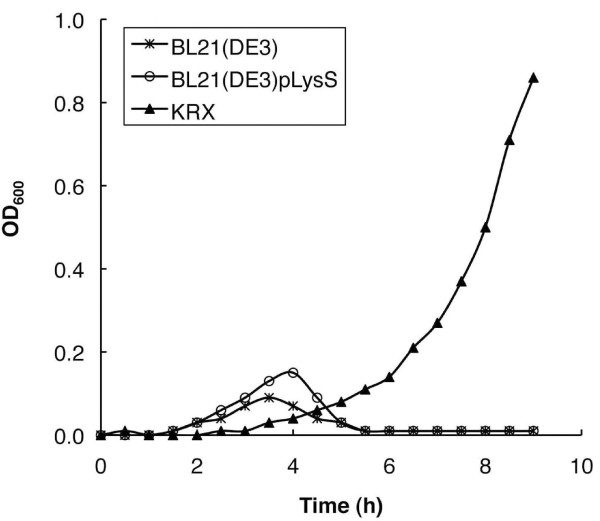
**Growth curves of various *E. coli *cells harboring the pIVEX-Danio pol β plasmid**. The growth of transformed bacterial cells (BL21(DE3) (cross), BL21(DE3)pLysS (open circle) and KRX (open square)) was performed in LB medium at 30°C. The OD**_600 _**was recorded at 30 min intervals.

*Danio *pol β was expressed as a C-terminal His-tagged fusion protein, using the pIVEX 2.3d expression system, in *E. coli *BL21(DE3), BL21(DE3)pLysS and KRX cells. After transformation into bacterial cells and picking the resultant colonies, bacterial cell growth was monitored by measuring the OD_600 _of the culture at 30 min intervals for 9 h without inducers (IPTG or rhamnose). Figure 2 shows that the growth of transformed BL21(DE3) and BL21(DE3)pLysS ceased at 3.5 and 4.0 h, respectively; however, the growth of transformed KRX continued throughout the monitoring period, although growth rate of KRX was slower than that of BL21(DE3) or BL21(DE3)pLysS. We also tested cell growth with pIVEX-rat pol β for comparison with pIVEX-*Danio *pol β. No inhibition of growth was observed using BL21(DE3)pLysS containing pIVEX-rat pol β whereas the weak growth inhibition for transformed BL21(DE3) cells was observed as a growth delay (data not shown). Growth inhibition might be caused by the adverse effects of leaky expression of *Danio *pol β in the bacterial cells. The *lac*I gene and its associated operator are not contained within pIVEX; therefore, when using pIVEX, transcription of the T7 RNA polymerase gene in the host chromosome at the *lac*UV5 promoter, and expression of recombinant protein at the T7 promoter are not controlled in BL21(DE3). Suppression of the basal expression in BL21(DE3)pLysS would also be difficult, because BL21(DE3)pLysS would not exert enough control of basal expression of T7 RNA polymerase, despite the T7 lysozyme encoded in the pLysS plasmid being an inhibitor of T7 RNA polymerase. The *E. coli *KRX cells, a derivative of the K12 strain, exerts precise control of T7 RNA polymerase gene transcription by the rhamnose promoter, permitting more exact control of recombinant protein expression. The basal protein production levels in KRX are 7- and 20-fold lower than in BL21(DE3) and BL21(DE3)pLysS, respectively [22]. Thus, we assume that precise suppression of basal expression of *Danio *pol β, which would be toxic to *E. coli*, is vital. For this reason, we selected pIVEX/KRX as the best plasmid/host combination to ensure stringent repression in the uninduced stationary state.

### Overexpression of *Danio *pol β

**Figure 3:**
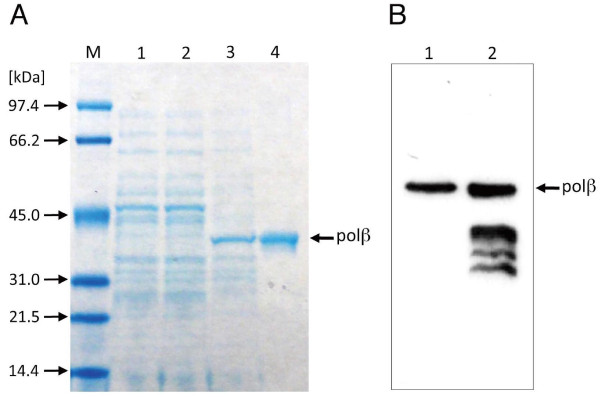
**Expression of *Danio *pol β *in vivo *and *in vitro***. (A) SDS-PAGE analysis of the proteins from ***E. coli ***KRX at each step of the expression and purification procedures. Lane M, molecular weight marker, Lane 1, uninduced cell extract (0 h); Lane 2, uninduced cell extract (12 h): Lane 3, induced cell extract (12 h); Lane 4, elute from Ni**^2+^**-chilete column chromatography. Proteins were stained with Coomassie brilliant blue (CBB). (**B**) Western blotting analysis of recombinant *Danio *pol β expressed *in vivo *(lane 1) and*. in vitro *(lane 2)

To investigate the properties of *Danio *pol β, we expressed the recombinant protein using pIVEX-*Danio *pol β in *E. coli *KRX. After induction with rhamnose, recombinant C-terminal His-tagged *Danio *pol β was purified in a soluble form from the cell extracts by column chromatography with Ni^2+^-NTA resin. The production yield of the recombinant protein, based on the ratio of the amount of the purified protein against the volume of the culture medium, was 7.4 μg/mL. The recombinant protein was analyzed by SDS-PAGE and Coomassie brilliant blue (CBB) staining. Figure 3A shows that His-tagged *Danio *pol β protein was expressed from pIVEX-*Danio *pol β in *E. coli *KRX. The recombinant His-tagged *Danio *pol β protein was detected as a 39.8 kDa band (Figure 3A).

To compare both *in vivo *and *in vitro *expression, we used the same pIVEX plasmid encoding *Danio *pol β. Figure 3B shows that *Danio *pol β was produced in a cell-free expression system using the pIVEX plasmid. From the western blotting analysis using anti-His_6_-antibody, proteolysis of *Danio *pol β occurred during *in vitro *expression (Figure 3B). As a result, bacterial expression was preferred to cell-free expression for *Danio *pol β.

### Enzymatic characterization of recombinant *Danio rerio *pol β

**Figure 4:**
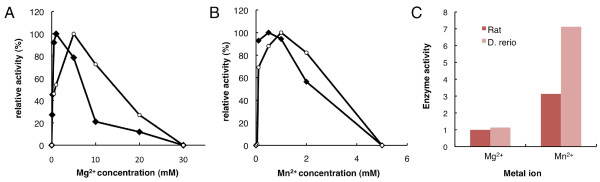
**Comparative analysis of metal ion activation for pol β of *Danio rerio *and rat**. Dependence on the Mg**^2+ ^**(A) or Mn**^2+ ^**(B) concentration of the pol β activity of ***Danio rerio ***(closed lozenge) and rat (open circle) were assayed in 0-30 mM Mg**^2+ ^**and 0-5 mM Mn**^2+^**. Comparison of the activity of ***D. rerio ***and rat pol β by Mg**^2+ ^**and Mn**^2+ ^**was performed at 1 mM MgCl**_2 _**and at 0.5 mM MnCl**_2 _**(C). The activity measured by the amount of incorporated dTTP. Each activity was normalized by the activity of rat pol β in the presence of Mg**^2+^**.

**Figure 5:**
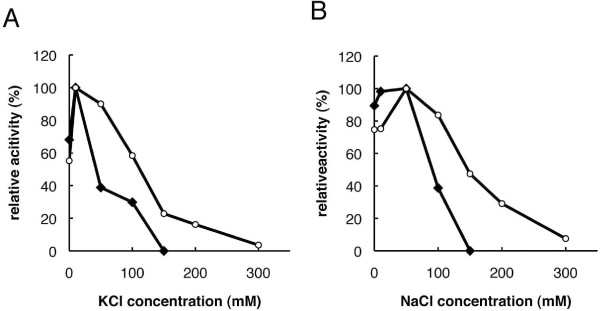
**Effect of salt on *Danio *pol β**. Effect of KCl (A) and NaCl (B) on the pol β activities of ***Danio rerio ***(closed lozenge) and rat (open circle). The activities of pol β were determined by the incorporation of dTTPs under standard assay conditions. Incubation was carried out at 30°C (***D. rerio***) or 37°C (rat) for 5 min.

We investigated the effects of the metal ions Mg^2+ ^and Mn^2+ ^on *Danio *pol β by measuring the enzyme activity in comparison with rat pol β. Figure 4A shows that a sharp peak at 1 mM Mg^2+ ^and a decrease in the activity was observed at concentrations higher than 10 mM Mg^2+ ^for *Danio *pol β; in contrast, a peak at 5 mM Mg^2+ ^with a broad decay was observed for rat pol β. Figure 4B shows that the optimal concentration of Mn^2+ ^was 0.5 mM for *Danio *pol β and 1 mM for rat pol β, followed by broadly reduced activity at higher concentrations. We also examined the effect of salt strength on pol β activity, using increasing concentrations of KCl and NaCl. Figure 5 shows that the concentrations permitting maximum activities of *Danio *pol β and rat pol β were the same for KCl and NaCl, at 10 mM KCl and 50 mM NaCl, respectively. No activity was observed at concentrations higher than 150 mM for both KCl and NaCl for *Danio *pol β. The optimum conditions of metal ion and salt concentration for *Danio *pol β were not significantly different compared with those for rat pol β. However, Figure 4C shows that *Danio *pol β activity with Mn^2+ ^was a dramatic 6.3-fold higher than that with Mg^2+^; in contrast, rat pol β activity with Mn^2+^was 3.1-fold higher than that with Mg^2+^. Thus, Mn^2+ ^is the optimal ion for pol β enzyme activity. However, Mn^2+ ^favors misincorporation of deoxynucleotides by pol β, compared with Mg^2+^. This agrees with the observation that Mn^2+ ^is known to relax base-pairing specificity and to increase tolerance for substrates compared with Mg^2+ ^for many polymerases [23]. For such reasons, Mg^2+ ^was used as the metal ion in subsequent experiments.

**Figure 6:**
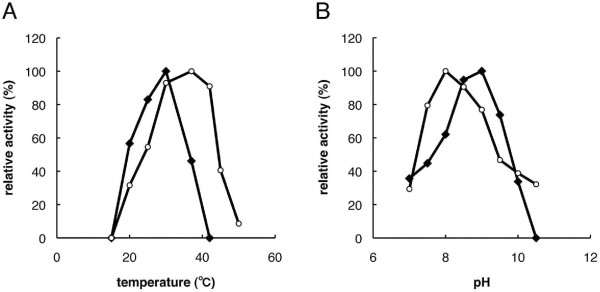
**Apparent temperature and pH optima of *Danio rerio *pol β**. Effect of temperature (A) and pH (B) on the pol β activities of ***D. rerio ***(closed lozenge) and rat (open circle). The activities of pol β were determined by the incorporation dTTPs under standard assay conditions. (A) Effect of pH on pol β activity was carried out at various pHs from 7.0-10.5. Incubation was carried out at 30°C (***Danio rerio***) or 37°C (rat) for 5 min. (B) Effect of temperature on pol β activity was carried out at various temperatures: 15-42°C (***Danio rerio***) or 15-50°C (rat).

We measured the pH dependence of *Danio *pol β in the range between pH 7.0 and pH 10.5. Figure 6Ashows peaks at pH 9.0 and 8.0 for *Danio *pol β and rat pol β, respectively, although both enzymes showed activity in a rather broad pH range. The optimum pH in the alkaline range was similar to other eukaryotic pol β enzymes. *Danio *pol β lost activity at pH 10.5.

We then examined the activity of *Danio *pol β at various temperatures, from 15°C to 50°C, and compared it with the rat polymerase activity. Figure 6B shows that *Danio *pol β was most active at 30°C and decreased by 50% at 35°C compared with the highest activity. *Danio *pol β was relatively sensitive to temperature compared with rat pol β. Furthermore, the optimum temperature of *Danio *pol β was lower than that of rat pol β (37°C). This observation corresponds to the lower temperature generally required by fish compared with the warmer body temperatures of humans and rats [11,12]. The fish and frog pol β proteins contain the P63D variation compared with mammalian pol β proteins. This difference might represent an important evolutionary mutation between cold-blooded and homeothermic organisms [12]. Indeed, *Danio *pol β also has the P63D variation (Figure 1).

### Kinetic assays of *Danio *pol β

We investigated the kinetic constants *K*_m _and *k*_cat_, and the catalytic efficiency *k*_cat_/*K*_m _for *Danio *pol β in the presence of either Mg^2+ ^or Mn^2+^. Table 1 summarizes the calculated *K*_m_, *k*_cat_, and *k*_cat_/*K*_m _of *Danio *pol β. Measurements for each dTTP concentration were performed with Mg^2+ ^or Mn^2+^. From Hanes-Woolf plots, the kinetic parameters *K*_m _and *k*_cat _for the analyzed substrate were derived, and their ratio (*k*_cat_/*K*_m_) representing the efficiency of substrate utilization was calculated. As summarized in Table 1, in the presence of Mn^2+^, *K*_m _decreased by 11.1-fold compared with that with Mg^2+^, whereas the *k*_cat _values were similar, resulting in a significant increase (8.6-fold) in *k*_cat_/*K*_m_. The decrease in *K*_m _represents an increased dNTP binding affinity. Mn^2+ ^undergoes ligand exchange approximately 100 times faster than Mg^2+^, although Mn^2+ ^serves as an excellent metal surrogate for Mg^2+ ^because of its similar ionic radii and coordination geometries [24-26]. This relatively tight binding of Mn^2+ ^to the dNTP binding site might increase in the activity. In contrast, a mechanism of nucleotide selectivity may depend on ligand exchange [27]. Thus, Mn^2+ ^could act not only as an enhancer for catalytic efficiency but also as a potent mutagen [23]. The large difference in *k*_cat_/*K*_m _when substituting Mn^2+ ^for Mg^2+ ^may indicate that the fidelity of *Danio *pol β is reduced.

**Table 1:** Kinetics of *Danio *pol β in the presence of Mg^2^^+ ^or Mn^2^^+^

Metal ion	Substrate	*K*_m _[μM]	*k*_cat _[min^-1^]	*k*_cat_/*K*_m _× 10^3 ^[μM^-1 ^min^-1^]
Mg^2+^	dTTP	14.69	0.74	0.05
Mn^2+^	dTTP	1.32	0.58	0.43

## Conclusions

We have succeeded in overproducing, purifying, and characterizing *Danio *pol β. Using a suitable plasmid/host combination, *Danio *pol β encoded by the *in vitro*/*in vivo *compatible pIVEX plasmid was expressed in *E. coli *KRX. This combination was able to tightly suppress leaky expression of *Danio *pol β, which is a toxic protein for bacterial cell growth. The pIVEX/KRX combination required only one antibiotic, unlike previous work using two antibiotics because of the requirement for an extra plasmid, such as pLysS. This combination also forms an important tool for proteomics and structural biology, with its potential to express a variety of (growth toxic) recombinant proteins in both *in vitro *and *in vivo*. Furthermore, we performed extensive biochemical characterization of *Danio *pol β, which revealed its sensitivity to metal ion activators, its optimum salt concentration, alkaline pH optimum, and low temperature optimum. From the kinetic assays, we found that substituting Mn^2+ ^for Mg^2+ ^resulted in 8.6-fold higher catalytic efficiency (*k*_cat_/*K*_m_). Mn^2+ ^is considered a potential mutagen, and further experiments to investigate the sensitivity and fidelity with metal ion activators for *Danio *pol β are underway. Moreover, we characterized pol β from the *D. rerio *fish model. Pol β is the smallest polymerase in eukaryotes, and it can be regarded as a basic model of various DNA polymerases. Our characterization of pol β from a model fish organism contributes to the study of the function and evolution of DNA polymerases.

## Methods

### Construction of the pIVEX-*Danio *pol β expression plasmid

A 1019-bp cDNA sequence of *Danio *pol β [DDBJ: AY648826] was artificially synthesized and codon-optimized for *E. coli *expression (Operon Biotechnologies). To clone the cDNA into pIVEX 2.3d vector (5 Prime), which expresses the protein with a C-terminal His_6_-tag, the synthesized cDNA was amplified by PCR using primers designed against the 5' and 3' conserved region of the cDNA, and the high fidelity Phusion DNA polymerase (Finnzyme). An additional 15 bp was included in each primer, which corresponded to upstream and downstream bases of the pIVEX 2.3d vector to enable ligation-independent In-Fusion cloning (Clontech). The primers were: forward primer 5'-AGG AGA TAT ACC ATG AGC AAA CGT AAA GCC CCT-3' and reverse primer 5'-ATG AGA ACC CCC CCC TTC TGA GCG ATC TTT TGG-3'. The underlined regions represent the *Danio rerio *pol β-specific sequence. The PCR products were separated on a 1% agarose gel and the band corresponding to the expected cDNA coding sequence was isolated. The gel-isolated DNA fragment was purified by ethanol precipitation and inserted into linearized pIVEX vector, which was digested at the multiple cloning site by *Nco*I and *Sma*I, using the In-Fusion system (Clonetech), according to the manufacturer's instruction. The integrity of the nucleotide sequence of the constructed plasmid was confirmed by DNA sequencing.

### Overexpression and purification of *Danio rerio *pol β

Plasmid pIVEX-pol β was used to transform *E. coli *KRX (Promega), BL21(DE3) (Nippon Gene), and BL21(DE3)pLysS (Novagen) cells for expression of *Danio *pol β. Transformed cells were plated and cultured on LB plates containing ampicillin (100 μg/ml) at 37°C overnight. Individual bacterial colonies were grown overnight in 5 ml of LB medium supplemented with ampicillin (100 μg/ml) at 30°C to a cell density of 0.6 OD_600_. During this pre-culturing, the OD_600 _value was recorded at 30 min intervals with an optical density monitor (Taitech). The next day, a fresh 1% inoculum was added into 200 ml of LB medium containing 100 μg/ml ampicillin at 25°C until an OD_600 _of approximately 0.8 was reached. At this point, 1% rhamnose (Promega) was added to the growth culture of KRX *E. coli *containing pIVEX-pol β for induction of recombinant protein expression. Cells were harvested by centrifugation at 3300 × *g *for 10 min, gently suspended in 20 ml lysis buffer (300 mM KCl, 50 mM KH_2_PO_4_, 5 mM imidazole, pH 8.0) and sonicated on ice (20 times, 30 s). After centrifugation at 5800 × *g *for 30 min, the soluble fraction was filtered through a 0.45 μm pore-membrane (Millipore). Recombinant protein was purified and its buffer was exchanged using the Profinia protein purification system (Bio-Rad). Recombinant protein in the soluble fraction was applied to an Ni^2+^-NTA affinity chromatography column with 1 ml column volume (Bio-Rad) previously equilibrated with wash buffer 1 (50 mM potassium phosphate, pH 8.0, 300 mM KCl, 5 mM imidazole). The column was allowed to equilibrate with the lysate and the unbound protein was eluted from the column with six column volumes of wash buffer 1, followed by elution of weakly bound protein with six column volumes of wash buffer 2 (50 mM potassium phosphate, pH 8.0, 300 mM KCl, 10 mM imidazole). Tightly bound fusion protein was eluted with three column volumes of elution buffer (50 mM potassium phosphate, pH 8.0, 300 mM KCl, 250 mM imidazole). The eluted protein was applied to a gel chromatography column (Bio-Rad) previously equilibrated phosphate buffer saline (137 mM NaCl, 2.7 mM KCl, 4.3 mM Na_2_HPO_4_, 8.1 mM KH_2_PO_4_, pH 7.4) for desalting of the elution buffer. The purified protein was concentrated at ~2 mg/ml by ultrafiltration using an Amicon Ultra filter (MWCO: 10 K, Millipore) by centrifugation for 30 min at 8000 × *g *at 4°C. The same volume of glycerol was added to the concentrated sample solution to make an approximately 1 mg/ml stock sample solution in 50% (v/v) glycerol for storage at -80°C. The production yield of the recombinant protein was evaluated by determining the amount of the purified protein against the volume of the culture. The amount of the purified protein was determined using the Bradford method. One milliliter of 1:5 diluted dye solution (Bio-Rad) was added to 100 μl of the purified protein and standards protein of bovine serum albumin. After incubation for 5 min at room temperature, an absorbance at 595 nm was measured. The amount of purified protein was determined by measuring the absorbance and referring to a calibration curve generated using the standard protein. Recombinant rat pol β was expressed and purified from *E. coli *as described by Date *et al*. [28]. Cell-free expression of *Danio *pol β and rat pol β was performed in RTS100 *E. coli *HY (5 Prime), as described in the instruction manual. Recombinant protein production was carried out in a 50 μl volume with 0.5 μg of pIVEX-pol β plasmid for 6 hours at 30°C. The expressed proteins from both *in vivo *and *in vitro *extracts were examined by SDS-polyacrylamide gel electrophoresis and subsequently detected by Coomassie brilliant blue staining and western blotting.

### DNA polymerization assay

The template and digoxigenin (DIG)-labeled primer used in the activity assay were purchased from Sigma-Aldrich. The primers were labeled with DIG at the 5' end for subsequent detection in Southern blotting. The DIG-primer, 5'-GGA GGA TGG CAG CGT GAG GG-3', annealed to the templates (5'-AAA AAC CCT CAC GCT GCC ATC CTC C-3') at a 1:1.2 molar ratio when heated at 95°C for 5 min and then at 65°C for 20 min, followed by immediate cooling on ice. The prepared primer-template complex (5 μM) was stored on ice until required.

Reactions for determining *Danio *pol β activity were performed in a 10 μl reaction mixture, containing 50 mM Tris-HCl (pH 8.8), 1 mM dithiothreitol (DTT), 100 μg/ml bovine serum albumin (BSA), 100 mM KCl, 1 mM MgCl_2_, 0.5 μM primer-template, 10 μM dTTP, and 0.1 mg/ml *Danio *pol β. The reaction mixtures were incubated at 30°C for 5 min and the reaction was terminated by the addition of 7 μl stop solution (95% formamide, 20 mM ethylenediaminetetraacetic acid, 0.05% bromophenol blue, and 0.05% xylene cyanol FF).

To compare the activity of *Danio *pol β with rat pol β, the reaction of rat pol β activity was performed in a 10 μl reaction containing 50 mM Tris-HCl (pH 8.0), 2 mM DTT, 150 mM KCl, 5 mM MgCl_2_, 0.5 μM primer-template, 10 μM dTTP and 0.1 mg/ml rat pol β. The reaction mixture was incubated at 37°C for 5 min, and 7 μl of stop solution was added to quench the reaction.

Products were analyzed by sequencing gel electrophoresis. Each quenched reaction mixture was incubated at 95°C for 5 min, and then analyzed by electrophoresis under denaturing conditions [12% acrylamide, 7 M urea, and sodium taurine (ST) running buffer] for 2.5 h with the power supply fixed at 40 W during electrophoresis [29,30]. Following electrophoresis, the gel was contact-blotted onto a positively charged nylon membrane (Roche) for 45 min and then cross-linked (200 mJ/cm^2^) using light at a wavelength of 254 nm. The membrane was incubated in a blocking buffer [0.1 M maleic acid, 0.15 M NaCl pH 7.5, 1% (w/v) blocking reagent; Roche] on a rocking platform for 30 min. The blocked membrane was incubated with anti-DIG-AP Fab fragments (Roche) diluted 1:50,000 in blocking buffer for 60 min. The membrane was washed three times with wash buffer (0.1 M maleic acid, 0.15 M NaCl, pH 7.5, 0.3% Tween 20) for 20 min each. The membrane was then incubated in detection buffer (0.1 M Tris-HCl pH 9.5, 0.1 M NaCl) on a rocking platform for 20 min. Finally, the membrane was incubated with CDP Star (Roche) at a 1:500 dilution in detection buffer for 10 min. The membrane was exposed to X-ray film (Fuji Film) in the dark and the film developed in a medical film processor (FPM100; Fuji Film).

### Kinetic assays of *Danio rerio *pol β

Kinetic Incorporation of dTTP (Takara) by pol β of *Danio rerio *or rat was performed in the same reaction buffer and temperature (30°C) that was used for the respective DNA polymerization assay in a total volume of 10 μl containing 0.5 μM of the primer-template complex. The concentrations of dTTP use were 3.75, 5, 7.5, 10, 15, and 20 μM. The reaction mixture was incubated for 5 min. The kinetics of dTTP incorporation were determined using sequencing gel assay [31,32]. The velocity of the reaction, *v *(amount of extension products per minute), was calculated from the ratio of the amount of extended products to the total amount of primer in each lane. The *K*_m _and the maximum velocity (*V*_max_) for dTTP incorporation were calculated using least-square fitting from a Hanes-Woolf plot of [dTTP]/*v *against [dTTP]. The *K*_cat _was calculated by dividing *V*_max _by the enzyme concentration. The amount of extended product was estimated from the intensity of each band following subsequent sequencing electrophoresis analysis with GelPro Analyzer software (Media Cybernetics).

## Competing interests

The authors declare that they have no competing interests.

## Authors' contributions

TI carried out all experiments except the DNA sequencing and the cell-free expression and drafted the manuscript. NY discussed on the molecular cloning studies and the results obtained. MI discussed on the results obtained and helped to draft the manuscript. KH conceived the study, and participated in its design and coordination and helped to draft the manuscript. All authors read and approved the final manuscript.
